# Hospitals of To-Day

**Published:** 1911-04-22

**Authors:** 


### HOSPITALS OF TO-DAY.

### II. WESTMINSTER HOSPITAL AND ITS SITE.

The history and progress of Westminster Hospital have been more influenced by its site than probably has been the case with any other London institution. Its present position and prospects are determined in a unique way by the size and situation of the land on which the present building was constructed in 1834. This land is only three-quarters of an acre in extent and stands, as everyone knows, at the heart of London. Church and State meet at its very doors. The result of this has been that more money has been spent in improving the existing buildings than they could ever have cost themselves, and at the same time the growth and modernisation of the institution have been limited and controlled by the impossibility of extension. Westminster Hospital has never been able to add one cubit to its stature, owing to the immense value of land on either side. The difficulties which this enforced limitation has caused to an institution with a great reputation to foster, and with an ever-changing standard of efficiency to maintain, are obvious to anyone who cares to walk round the building.

There have been compensatory advantages, however. Indeed, it would seem as if a *genius loci* still hovered in Westminster and from the magic of that name contrived to make good in other ways the limitations of the site in the matter of size.

A glance at the latest report illustrates this. It states that the number of out-patients is again decreasing at the hospital. This is nowadays a sufficiently rare event to be worthy of notice, and its importance in the case of a hospital which is precluded from extension is clear enough. It is true that even now nearly 20,000 persons are treated in the out-patient department at Westminster, but there is all the difference in the world between this number as the highest limit and as intermediate to a still larger figure.

It would seem, again, that the site were working on the hospital's behalf, for the cause of this decrease in numbers is due to the value of the immediate neighbourhood, owing to which a congeries of small streets has been demolished to make way for such buildings as the Wesleyan Centenary Hall. A comparative analysis of the two classes of which the out-patients are composed shows that the casualties have diminished at more than three times the rate of the out-patients. But it is the latter alone who come from a wide area; the casualties, of course, are brought from close at hand. In this way the one hospital which was unable to meet the general demand for increased out-patient accommodation has been spared the necessity of its provision.

How have the hospital's finances been affected by its situation? It cannot be denied that Westminster Hospital, on the whole, has been fortunate in its financial history. Last year, it is true, there was a deficiency of £4,590, but a hospital's financial condition must be judged by a longer period than one year, and for the last ten years or so there has generally been a surplus. No doubt its name—in other words, its site—has stood it in good stead, and has helped to explain why the hospital has been advertised so little. Westminster is a name which needs no advertising, and Sir J. B. Wolfe Barry, the distinguished chairman, has done his work and pressed his institution's claims in ways less publicly obtrusive. Moreover, at recurring intervals the hospital possesses a means of raising money which is peculiarly its own. This means, of course, is the commercial value of its position opposite the Abbey at the time of the Sovereign's Coronation. When any great event takes place in the Abbey, Westminster Hospital has the best view of the arrival and departure of all who form part.

Once again, it may be pointed out, the site will come to the rescue, and the deficiency of last year already noted, which was increased by the sudden drop in the amount received from legacies, is likely more than to be paid by means of the sum which the seats that the hospital will provide to view the Coronation will produce. The procedure and its results this year may be gathered from what happened in 1902. Then, as now, half a dozen stands were erected on the east and south fronts of the hospital, which are exactly opposite the Royal entrance to the marshalling hall. These were taken so quickly that it was hoped to realise £12,000, but the unforeseen postponement of the Coronation caused a loss of nearly half that sum. The seats were sold on the condition that if the ceremony did not take place the booking-fee should be returned on request, less a percentage to meet the expenses, which were necessarily increased by the postponement. This year the same procedure will be adopted, and, in order to give the supporters of the hospital and their friends the first chance, no public advertisement concerning the stands was issued till April 1. As Westminster Hospital is undoubtedly the finest position in London from which to see the Coronation procession, it is not surprising that most of the seats, which are protected from ill weather, have been sold. It is likely, then, that this year again Westminster Hospital will have a clear balance-sheet.

The hospital, therefore, continues its quiet progress under the unique circumstances which have conditioned its existence. The reconstruction schemes, the laying of foundation-stones, the announcements of appeals for new buildings, have little place here. But, as an example of the up-to-dateness of its methods, Westminster was one of the first hospitals to employ an almoner, whose duties, let us add, are interpreted in no purely restrictive sense. Many medical or surgical convalescents have been assisted by this official.

London hospitals serve a larger area than the Metropolis; their position in the capital has not to be regulated only by the expansion of London, and it is to be hoped that Westminster Hospital will remain a long time yet to enjoy the peculiar responsibilities and advantages of its site by the Abbey. The question of moving it, say, to Putney, will arise no doubt one day, but that is not a course likely to be considered seriously at present.

